# Chlorinated Pools: Bernard et al. Respond

**Published:** 2007-05

**Authors:** Alfred Bernard, Sylviane Carbonnelle, Marc Nickmilder

**Affiliations:** Department of Public Health, Catholic University of Louvain, Brussels, Belgium, E-mail: alfred.bernard@uclouvain.be

We are pleased to respond to Eggleston because this offers us the opportunity to respond to some of the criticisms that have been formulated since we originally proposed the pool chlorine hypothesis (Bernard et al. 2003).

First, the divergent effects of chlorine [described in our recent study (Bernard et al. 2006)]—when this chemical is used to clean surfaces or to sanitize recreational water—are not inconsistent. Chlorine is a nonspecific biocide, and there are clearly situations in which the beneficial effects of this agent need to be balanced against its possible adverse effects. As we explained in the “Discussion” of our recent articles (Bernard et al. 2006; Nickmilder et al. 2007), exposure conditions are radically different when children live in a house cleaned with bleach compared with when they attend an indoor chlorinated pool. When a house is cleaned with bleach, children are not likely to be exposed to high concentrations of chlorine gas or trichloramine because they are not directly involved in the cleaning tasks. In that situation, the balance for children—but not necessarily for people doing the cleaning—is clearly in favor of the beneficial effects of chlorine from a decreased risk of asthma and respiratory allergy (Bernard et al. 2006; Martyny et al. 2005; Nickmilder et al. 2007). In contrast, when attending an indoor pool, children are directly in contact with the chlorination products that they actively inhale as gases, aerosols, or even water. It can be argued that the time children spend in a swimming pool is limited, but we should not forget that chlorine-based chemicals are rapidly acting oxidants, a property essential to their efficacy.

Eggleston raises the issue of a possible confounding between age and lifetime cumulative pool attendance (CPA). However, because our study (Bernard et al. 2006) focused on children in 5th and 6th grades, there is little variation in age (range 10–13 years), explaining why age did not emerge as a predictor of the outcomes (Table 1) and also why it did not vary across CPA categories (analysis of variance, *p* = 0.35). Eggleston states that cases of asthma cannot be allocated to the CPA categories of our Figure 1, but this is because he has misinterpreted the way these categories were constructed. Subjects were not divided into quartiles but into predefined categories of increasing CPA. If numbers of subjects included in each category approximate those of quartiles, this is no more the case when each category is further divided according to the total serum IgE. The reason for this is given in Figure 3, which shows that the proportion of children with higher serum IgE gradually decreases as CPA increases.

We agree with Eggleston that the exhaled nitric oxide (eNO) test is not a specific measure of airways inflammation in asthma. Rhinitis is a potential confounder that we took into account by adjusting the odds ratios (ORs) for the sensitization to aeroallergens, including house dust mites, the most frequent allergen in allergic rhinitis. We did not retain medication for asthma or allergy in the final analysis because of the strong collinearity of this factor with some outcomes, such as doctor-diagnosed asthma. However, adding medication to the list of possible predictors did not abolish the association between eNO and CPA (OR, 1.32; 95% confidence interval, 1.09–1.60). We also found no confounding by viral infections, which is not surprising because children seriously affected by a respiratory illness were absent from schools at the time of examination.

We used objective measures whenever possible in our study (Bernard et al. 2006), but in order to derive predictors such as CPA, we had no choice but to use the information provided by parents and school directors (for compulsory pool attendance at school). We believe that the strong associations found in our study should not be dismissed as having arisen by bias or insufficient adjustment. However, what makes us increasingly confident in our observations is their reproducibility. The findings reported in our study (Bernard et al. 2006) confirm earlier observations (Bernard et al. 2003), and a new larger study on adolescents, just completed, again brings to light quite strong associations between different indicators of asthma and CPA, especially among atopic children (Bernard et al., unpublished data).
